# Changes of microRNAs-192, 196a and 203 correlate with Barrett's esophagus diagnosis and its progression compared to normal healthy individuals

**DOI:** 10.1186/1746-1596-6-114

**Published:** 2011-11-17

**Authors:** Pavla Luzna, Jan Gregar, Ivo Uberall, Lenka Radova, Vlastimil Prochazka, Jiri Ehrmann

**Affiliations:** 1 Department of Histology and Embryology, Faculty of Medicine and Dentistry, Palacky University and University Hospital, Hnevotinska 3, 775 15 Olomouc, Czech Republic; 2 Department of Internal Medicine II - Gastroenterology and Hepatology, Faculty of Medicine and Dentistry, Palacky University and University Hospital, I.P. Pavlova 6, 779 00 Olomouc, Czech Republic; 3 Department of Clinical and Molecular Pathology, Faculty of Medicine and Dentistry, Palacky University and University Hospital, Hnevotinska 3, 775 15 Olomouc, Czech Republic; 4 Institute of Molecular and Translational Medicine, Faculty of Medicine and Dentistry, Palacky University and University Hospital, Hnevotinska 3, 775 15 Olomouc, Czech Republic

**Keywords:** microRNA, Barrett's esophagus, real-time PCR

## Abstract

**Background:**

Barrett's esophagus (BE) is a disease with a rising prevalence in western countries probably due to the unhealthy lifestyle. In significant number of cases it develops to esophageal adenocarcinoma. Two decades ago, important gene regulators (microRNAs) were discovered and their attendance in the process of malignant transformation was demonstrated (e.g. miR-192, 196a, 203). Our aim was to select the patients with the increased risk of malignant transformation before the cancer develops.

**Methods:**

71 patients with BE disease were selected, slides from FFPE blocks were prepared, the lesions were microdissected and a qPCR relative expression analysis for selected microRNAs (generally known to be connected with malignant transformation process) was carried out.

**Results:**

We demonstrated unequivocal statistically significant upregulation of two microRNAs (miR-192, 196a) and downregulation of miR-203 and positive miR-196a correlation with progression from intestinal metaplasia to adenocarcinoma compared to normal individuals.

**Conclusions:**

We hypothetize that there do exist changes of selected microRNAs which can undoubtedly distinguish the patients with BE from normal healthy individuals.

## Background

### Barrett's esophagus

Barrett's esophagus (BE) is the gastrointestinal disorder termed after the physician Norman Barrett in the 1950s [1]. It is an infliction of a distal part of the esophageal mucosa. In physiological state, esophageal mucosa is lined by squamous stratified nonkeratinized epithelium. However, by the influence of several agents the squamous epithelium is being replaced by the columnar one, the phenomenon known as metaplasia [2]. Subjectively, the patient with BE can suffer from heartburn, eructation or nausea, nevertheless, sometimes BE can be asymptomatic [3,4]. Grossly, BE is classified on the basis of its length: long segment (afflicted part of the esophagus is longer than 3 cm), short segment (shorter than 3 cm), and ultra-short segment (which is not actually observed by the endoscopic examination) [5]. Microscopically, Barrett's esophagus was defined by the presence of goblet cells. Today, however, it is accepted that there are three histologic subtypes of columnar epithelium which are found near to the distal esophagus [6], whereas only one of them is characterized by goblet cells presence. The first type is the junctional type secreting mucus, the second one is the gastric type of columnar epithelia with the presence of parietal and chief cells and the third one called „specialized intestinal metaplasia“ with the goblet cells presence which is the most proximal to the squamous epithelium [7].

BE can progress through dysplasia stages (low grade and high grade dysplasias) and even to the esophageal adenocarcinoma (EAC) [8]. Thus, Barrett's esophagus presents a sequential model of carcinogenesis. The attention to the EAC diagnosis is outright because of its rising prevalence in western countries [9] and its extremely high mortality oscillating about 85% within 5 year period [10].

### MicroRNAs

MicroRNAs form a large group of 18-22 nt short single-stranded RNAs with a high potential of gene expression regulation [11]. MicroRNAs were discovered as late as in 1993 by Victor Ambros and his colleagues in the worm *Caenorrhabditis elegans*. Successively, microRNAs were revealed across the plants and other animals including *Homo sapiens*. Nowadays, 1100 diverse microRNAs were identified in humans [12]. Due to their size and properties, they can create complementary bounds with high amount of mRNAs and therefore block the process of translation or to degrade the mRNA. It is estimated that 30% of all human genes are regulated by them.

It is important and fundamental to note, that their up and downregulation is correlated to various disorders including malignant tumors [13,14]. It is presumed that the huge group of microRNAs is the potential target for therapy of all sorts of diseases including malignant ones [15]. There are hundreds of studies monitoring levels of hundreds of microRNAs in malignant versus healthy tissues and hundreds of studies focused on the functional role of these molecules in the process of carcinogenesis [13,14,16-18].

Therefore the aim of this study was to identify a combination of selected microRNAs levels of which (compared to normal healthy individuals) would definitely characterize a patient with Barrett's esophagus diagnosis, even in spite of the fact that the histological examination is not clear. Further, we wanted to find out if a specific microRNA which would reflect the grading of the progression does exist. All the previous studies performed used usually frozen tissue samples and the number of the examined patients was low [19-22]. Nevertheless, archives of departments of pathology are offering enough biological material for the retrospective studies and it was confirmed that FFPE tissues are suitable for routine microRNA levels determination [23-27].

It is well known that the process of carcinogenesis is generally connected to major important genes involved in e.g. cell cycle, proliferation or inflammation. That's why we explored the miRBase and http://microRNA.org databases and searched for the concrete microRNAs which could impact the target genes from the selected groups. Due to our ongoing immunohistochemical studies we chose *IL-1beta *and *IL-8 *genes, revised the literature and we found out that they could regulate the expression of miR-21 resp. miR-203 as well. According to the literature, miR-192 is tightly connected to the digestive system carcinomas and miR-196a was found to have a potential to influence the cell cycle inhibitor *p27*, and *p27 *is mentioned as a colorectal carcinoma progression marker and pancreatic ductal adenocarcinoma biomarker, too. Simultaneously, we discussed our chosen miRNAs with present literature findings and finally decided to use four chosen microRNAs.

## Methods

### Tissue processing and staining

All the experimental and tissue handling approaches were approved by the Ethical Comittee of the University Hospital Olomouc. Bioptical specimens of esophageal mucosa were taken as a part of the regular endoscopic examination of the BE diagnosis by NBI (narrow band imaging) and AFI (autofluorescence). Immediatelly after the mucosa excision, the tissue was immersed into the Baker's solution and fixed for 24 hours. Consequently, after tissue processing it was embedded into paraffin. A routine H-E staining was performed and the grade of dysplasia was estimated. Consequently, a histochemical staining (PAS, Alcian blue) was performed according to the standard protocol.

### Laser capture microdissection (LCM)

All the methodical accesses were held under RNAse free conditions. We examined 71 patients diagnosed for BE (12 BE diagnosed macroscopically by NBI - microscopically not confirmed, 20 BE without dysplasia, 27 with low grade dysplasia and 12 with high grade dysplasia/adenocarcinoma). Since this is a retrospective study, the paraffin blocks originated from years 2006-2010. Paraffin blocks were cut for 5-6 μm thick slides which were mounted on the PET membrane-coated metal frame (before this the frame was exposed to 30 min of UV light, followed by poly-L-lysin coating in termostat for 30 minutes in 37°C). For better adhesion of the tissue the frames were put into the incubator for 30 minutes and 58°C. Concurrently, a routine H-E slide was prepared for better observation and orientation in the tissue architecture, because the slides used for microdissection have to be air-dried, thus cellular structures are worse recognizable.

The membrane coated slide with the mounted tissue was deparaffinized in two baths of xylene, 3 minutes each and consequently put into the 100% ethanol. Further, 95%, 75% and 50% ethanol solutions were used, 30 seconds each. Subsequently, the slides were stained by the cresyl violet solution for 1 minute, then washed by the rising concentrations of ethanol (50%, 75%, 95% and 100%) and finally washed in xylene, air dried and stored under the silicagel. The following LCM process was performed afterwards immediately.

The PET coated metal frame was turned around and placed on the basic histological slide and put into the microdissector. The requested tissue area was marked and dissected by the UV laser. Immediately after the dissection, the microdissected tissue was collected by the adhesive cap of the special eppendorf tube. We used the *mmi CellCut^® ^*(Molecular Machines & Industries, Zürich, Switzerland) microdissection instrument.

### RNA extraction, multiplex reverse transcription, preamplification step

Immediately after LCM, the eppendorf tube with the tissue was filled by 100 μl of digestion buffer, freezed at -80°C or RNA was promptly isolated. Prior to the RNA isolation, protease digestion was performed for 3 hours in 50°C and 15 minutes for 80°C. RNA including the small microRNA fragments was extracted by the RecoverAll™ Total Nucleic Acid Isolation Kit for FFPE (Ambion/Applied Biosystems/Life Technologies; Carlsbad, CA, USA) according to manufacturer's protocol. The elution volume was 60 μl and it was consequently spinned in a vacuum concentrator for 1,5 hour to reduce the final volume to approximately 20 μl. The concentration of RNA was measured by the NanoDrop spectrophotometer (Wilmington, DE, USA) and the yield was moving around 10 ng/μl. The isolated RNA was subjected to the reverse transcription and eventually stored at -80°C.

Every reverse transcription reaction contained the amount of 5 ng of total RNA isolated. The total volume of the reaction was 7,5 μl and it consisted of 3,5 μl of master mix (0,075 μl 100 mM dNTPs with s dTTP; 0,5 μl MultiScribe™ Reverse Transcriptase, 50 U/μl; 0,75 μl 10× Reverse Transcription Buffer; 0,095 μl RNAse Inhibitor, 20 U/μl; 2,08 μl DEPC water), all from TaqMan^® ^MicroRNA Reverse Transcription Kit (Applied Biosystems/Life Technologies; Carlsbad, CA, USA); 2,5 μl RNA and 1,5 μl RT stem-loop RT-primer pool (10 RT-primers, concentration 0,5× each: RNU44, RNU48, RNU66, RNU6B, U47, miR-16, miR-21, miR-192, miR-196a, miR-203) (all from Applied Biosystems/Life Technologies; Carlsbad, CA, USA). The reverse transcription reaction proceeded according to the protocol recommended by the manufacturer (30 minutes in 16°C, 30 minutes in 42°C, 5 minutes in 85°C). For the signal transduction the preamplification step was inserted according to [28].

### Real time PCR assay, PCR efficiency, endogenous control selection

The total volume of the qPCR reaction mixture was 10 μl. The mixture consisted of 5 μl of the TaqMan^® ^2× Universal Master Mix II No AmpErase UNG, 2 μl of nuclease free H_2_O, 0,5 μl of single TaqMan^® ^microRNA assay (all from Applied Biosystems/Life Technologies; Carlsbad, CA, USA) and 2,5 μl of the preamplification product. The amplification step was designed according to the manufacturer's protocol [95°C for 10 minutes, 45 cycles (95°C for 15 seconds, 60°C for 60 seconds)]. All the qPCR reactions were performed in the *LightCycler*^® ^480 System (Roche Applied Science, Penzberg, Germany).

For the efficiency of the qPCR reaction the FirstChoice^® ^Stomach RNA (Ambion/Applied Biosystems/Life Technologies; Carlsbad, CA, USA) was used because BE is a glandular type of metaplasia. Normal glands are found in stomach which is simultaneously the examined area during the esophageal endoscopy. So, the efficiency of PCR reaction should be verified in the glandular tissue.

The qPCR reaction for the chosen microRNA assay was adjusted for the 5 RNA template concentrations. The preamplification step was inserted as well. All the volumes were the same as for the regular qPCR reaction. After the reaction run, the efficiency was counted by the plotting the Cp value towards the log of the RNA concentration. The efficiency was presented by the slope of the axis.

Each qPCR reaction was performed for 6 potential endogenous controls (RNU44, RNU48, RNU66, RNU6B, U47, miR-16) according to the most cited reference genes in literature [20,29]. The most suitable two endogenous controls were assessed by the free downloadable application geNorm. The latter calculates a gene expression normalization factor for each tissue sample based on the geometric mean of a user-defined number of reference genes [30,31].

### Statistical analysis

The results of the study were processed by the biostatistician. The Kruskal-Wallis test with the multiple comparison was used for the p value formulation. In the regression relations (grading compared to other chosen parameters) the test of the coefficient significance in the regression model was used [32].

### Fold changes

The results of the experiments are presented as the x-fold change. It is the ratio of the relative gene expressions (relative expression of the examined gene in the sample and relative expression of this gene in the calibrator - normal healthy esophagus). The relative gene expression is the expression of the target gene divided by the expression of the reference (housekeeping) gene. The number of the fold expresses how many times is the relative expression of the target gene higher or lower than the relative expression of the same gene in the calibrator. The x-fold is expressed by formula [33]:

$$\text{ratio}=\frac{{\left({\text{E}}_{\text{Ref}}\right)}^{{\text{C}}_{\text{P}\;\text{sample}}}}{{\left({\text{E}}_{\text{target}}\right)}^{{\text{C}}_{\text{P}\;\text{sample}}}}÷\frac{{\left({\text{E}}_{\text{Ref}}\right)}^{{\text{C}}_{\text{P}\;\text{calibrator}}}}{{\left({\text{E}}_{\text{target}}\right)}^{{\text{C}}_{\text{P}\;\text{calibrator}}}}$$

cp = threshold point

E = efficiency of the qPCR reaction

sample = Barrett's esophagus in any grade

calibrator = normal healthy esophageal epithelium

On the basis of geNorm, reference genes RNU44 and U47 were used and all the x-fold changes are relativized to their geometrical mean.

## Results and discussion

**Figure 1:**
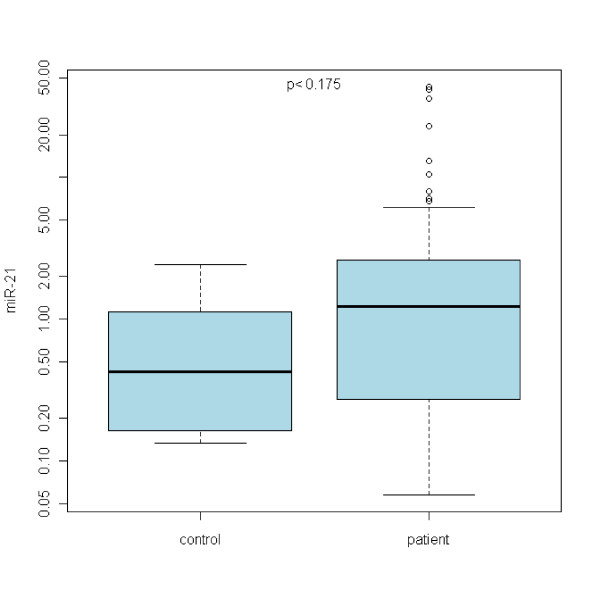
**miRNA-21 expression in normal healthy esophagus vs Barrett's metaplasia**. No statistically significant differences in miR-21 expression were observed.

**Figure 2:**
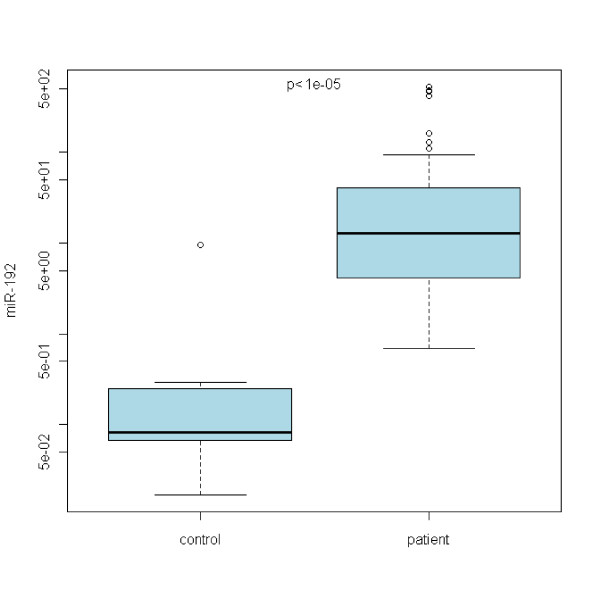
**miRNA-192 statistically significant upregulation in BE patients**.

**Figure 3:**
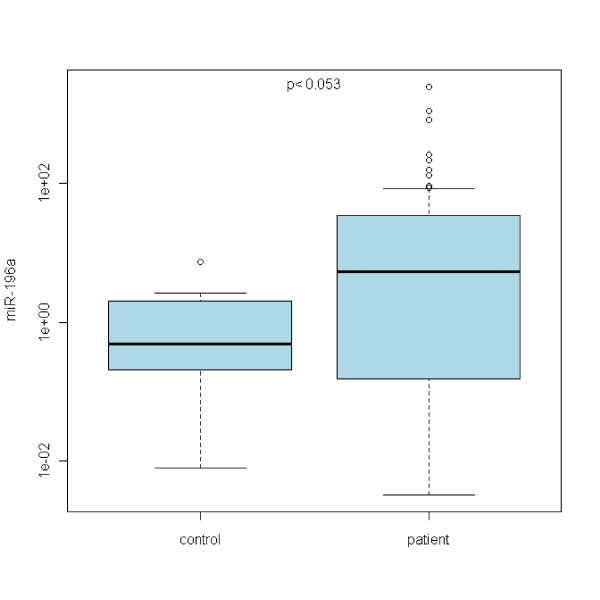
**miRNA-196a upregulation in BE patients is statistically significant**.

**Figure 4:**
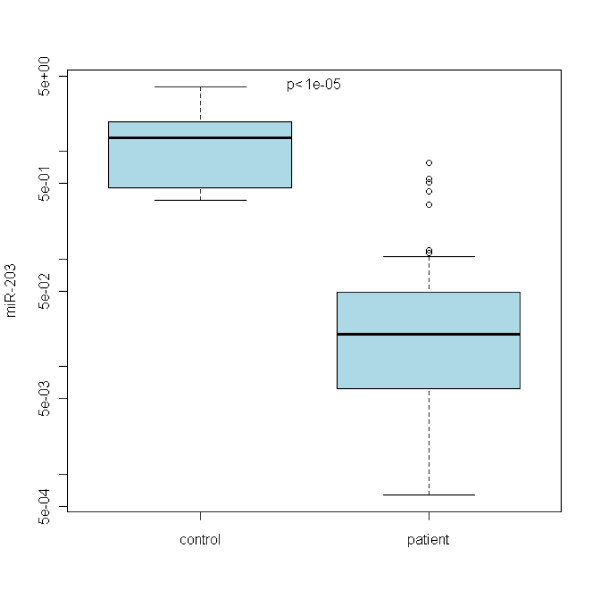
**Statistically significant downregulation of miR-203 in BE patients correlated to normal healthy esophageal tissue**.

**Figure 5:**
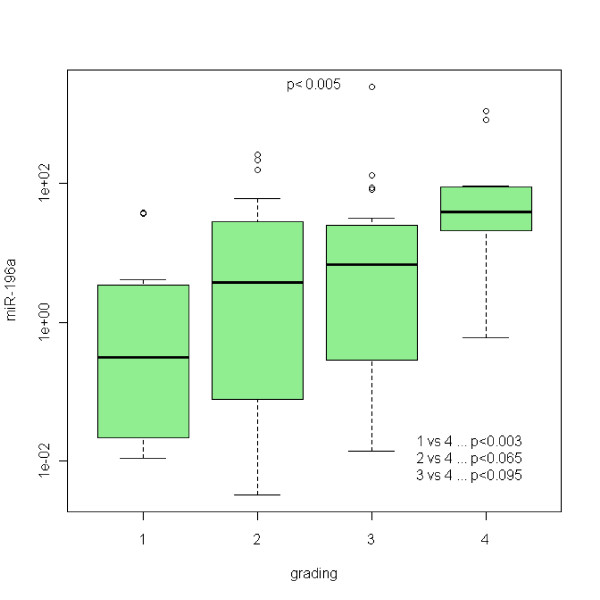
**Rising miRNA-196a upregulation in BE patients in NBI detected BE(1), BE(2), low-grade dysplasia(3), high-grade dysplasia/adenocarcinoma(4)**.

In the case of miR-21, no statistically significant differences between Barrett's esophagus and normal epithelia were acquired (see Figure 1). Refering to miR-192, there is a statistically significant upregulation of miR-192 expression in any grade of the disease with the p < 0,00001 (see Figure 2). miR-196a expression shows the upregulation as well with p < 0,05 (see Figure 3). On the other hand, miR-203 is definitely downregulated in the impaired tissue with the p < 0,00001 (see Figure 4). In the case of miR-196a, there is a statistically significant (p < 0,005) trend in relative expression dependent on the disease grading (see Figure 5). The results acquired from the analysis of disease grading did not confirm the dependency on sex, hiatal hernia, obesity, smoking, alcohol abuse, family anamnesis of any cancer, each of this risk factor being estimated separately (Pearsons Chi-Quadrat Test with Monte Carlo Simulations). Kruskal-Wallis Chi-Quadrat Test did not confirm the correlation of the grading versus age of patients as well as it did not confirm the correlation of the grading versus alcohol and smoking (every risk factor considered separately). However, correlation of grading versus hiatal hernia, obesity, smoking, alcohol abuse, family anamnesis (all parameters considered together) has confirmed that there are statistically significant parameters for alcohol (p < 0,046) and smoking (p < 0,065). Further, the correlation of grading to all the previous parameters plus sex and age shows statistically significant values of the influence of sex (p < 0,008), age (p < 0,065), smoking (p < 0,02), and alcohol abuse (p < 0,0045).

It is supposed that Barrett's metaplasia arises on the basis of chronic inflammation [34,35] which originates as a reaction to the gastroesophageal reflux disease (GERD). GERD occurs when the acid content of the stomach returns to the distal part of the esophagus. The continuous irritation of the esophageal squamous stratified nonkeratinized epithelium induces its replacement by the columnar one which is less mechanically resistant but it resists to the low pH of the esophageal microenvironment. Connection between BE and GERD was well established in the 1970s [6,36]. However, when there is GERD in the esophagus, BE must not be necessary present. GERD is not the only risk factor for development of BE. It is the presence of hiatal hernia, obesity (visceral fat), smoking or alcohol abuse as well. Moreover, men are three times more in danger than women. O'Riordan et al. [34] demonstrated that in the inflammation - BE - EAC sequence the levels of proinflammatory cytokines IL-8 and IL-1β increase. Thus, interleukins seem to be one possible target of the therapeutic intervention in the future. Another possible target of BE therapy could be the microRNAs - short single-stranded molecules with high potential of gene regulation.

It is well known that many microRNAs are deregulated in the connection with malignant progression. Changes in microRNA-196a expression are cited in connection with both pancreatic adenocarcinoma and breast carcinoma as well as in Barrett's esophagus. In combination with another miRNA (miR-217), miR-196a level can help distinguish between malignant and benign pancreatic tissue [37]. Moreover, in long-term survival adenocarcinoma patients (24 months), the levels of miR-196a are conversely correlated with survival [38]. As well as Mathé et al. [21], we also revealed that microRNA-196a is definitely overexpressed in Barrett's esophagus of any grade compared to normal healthy esophageal tissue, even the statistical analysis shows that the trend of the rising expression of this microRNA during the BE progression does exist. Moreover, compared to [20] (11 FFPE patients), we demonstrated this fact in our study with 71 patients in 4 gradings of BE.

In the case of miR-21, it was published, that six solid tumor tissues (lung, breast, stomach, prostate, colon, pancreas) where miR-21 is the only overexpressed microRNA do exist [39]. Feber et al. [19] describes the overexpresson of miR-21 and downexpression of miR-203 in his experiments with Barrett's esophagus as well as with esophageal adenocarcinoma. In the paper from Saini et al. [40], microRNA-203 is described as a anti-metastatic small RNA with the possible therapeutic intervention. Our results suggest the unequivocal miR-203 downregulation in BE, but non-unequivocal miR-21 expression. Compared to our study, Feber performed his experiments for 35 frozen specimens without the microdissection step. The same conclusion (for miR-21 and miR-203) can be find in the paper from Mathé et al. [22] who compared miRNA expression profiles of esophageal adenocarcinoma and squamous cell carcinoma to the adjacent noncancerous tissue pairs. In addition, Mathé et al. [22] describe the elevation of levels of miR-192 in esophageal adenocarcinoma. In our set of patients, we have come to the same conclusion.

Dealing with the possibility of getting the tissue samples through all stages of BE progression in one individuality brings controversial situation. We succeed in obtaining of tissue samples from all stages of BE progression in one patient's case, however we have to point out that the NBI technique, which we are using in the endoscopic examination helps to vizualize the architecture of the BE lesion and gives us the most precise picture of the stage of potential dysplasia and if there are any indications of proliferation to higher stage of BE dysplasia, it is therapeutically intervened. Our aim is not to let the patient to escalate to HGD/EAC stage which is very difficult to cure.

Most of the gene expression studies cited deal with relative expression data, but not all of them deal with the endogenous controls. Nonn et al. [28] use the normalization of the results to two chosen small nucleolar RNA RNU44 and RNU48, Maru et al. [21] use miRNA-16 as the reference gene. In his Application Note (Applied Biosystems/Life Technologies; Carlsbad, CA, USA) Wong et al., 2007 describe all the applicable endogenous controls suitable for microRNA data normalization, however with the necessity of verification. That's why in this paper six possible endogenous controls were chosen and the most stable couple of them was chosen for relative expression data calculation.

## Conclusions

In our study, we showed that there possibly do exist such combinations of microRNA expressions of which may be able to give us an information about the patient's status and possible disease progression. Expression of microRNA-196a was unequivocally confirmed as the Barrett's esophagus molecular marker with the rising trend through the disease progression. In combination with expression of microRNA-192 and microRNA-203 the patient could be independently diagnosed and his diagnosis could be confirmed in spite the fact that the BE diagnosis is not supported by the histological examination.

## List of abbreviations used

AFI: autofluorescence; BE: Barrett's esophagus; cp: threshold point; DEPC: diethylpyrocarbamate; dNTPs: deoxynucleotide triphosphates; dTTP: deoxythymidine triphosphate; E: efficiency; EAC: esophageal adenocarcinoma; FFPE: formalin fixed paraffin embedded; GERD: gastroesophageal reflux disease; H-E: hematoxylin-eosin; IL: interleukin; LCM: laser capture microdissection; miR: microRNA; mRNA: mediator ribonucleic acid; NBI: narrow band imaging; nt: nucleotide; PAS: periodic acid Schiff; PCR: polymerase chain reaction; PET: polyethylene terephthalate; qPCR: quantitative polymerase chain reaction; RNA: ribonucleic acid; RT: reverse transcription; UV: ultraviolet

## Competing interests

The autors declare that they have no competing interests.

## Authors' contributions

PL: participated in the design of the study, carried out the molecular studies, carried out histological stainings, JG: performed the endoscopical examination of patients, collected tissue samples and patient's history, IU: carried out the molecular studies, LR: carried out the statistical and correlation analyses, VP: conceived the study and participated in its design, collected tissue samples and patient's history, JE: conceived the study and participated in its design, evaluated endoscopical biopsies

All authors read and approved the final manuscript.

## References

[B1] Barrett N Chronic peptic ulcer of the oesophagus and „oesophagitis“Br J Surg 1950 30 175 182 10.1002/bjs.18003815005 14791960

[B2] Sternberg SS Histology for Pathology 1997 Philadelphia: Lippincott Williams & Wilkins

[B3] Halvorsen JF Semb BK The "Barrett syndrome" (the columnar-lined lower oesophagus): an acquired condition secondary to reflux oesophagitis. A case report with discussion of pathogenesis Acta Chir Scand 1975 141 683 687 1211042

[B4] Gaddam S Sharma P Advances in endoscopic diagnosis and treatment of Barrett's esophagus J Dig Dis 2010 11 323 333 10.1111/j.1751-2980.2010.00458.x 21091894

[B5] Reid BJ Li X Galipeau PC Vaughan TL Barrett's oesophagus and oesophageal adenocarcinoma: time for a new synthesis Nat Rev Cancer 2010 10 87 101 10.1038/nrc2773 20094044 PMC2879265

[B6] Spechler SJ Fitzgerald RC Prasad GA Wang KK History, molecular mechanisms, and endoscopic treatment of Barrett's esophagus Gastroenterology 2010 138 854 869 10.1053/j.gastro.2010.01.002 20080098 PMC2853870

[B7] Paull A Trier JS Dalton MD Camp RC Loeb P Goyal RK The histologic spectrum of Barrett's esophagus N Engl J Med 1976 295 476 480 10.1056/NEJM197608262950904 940579

[B8] Adler RH The lower esophagus lined by columnar epithelium. Its association with hiatal hernia, ulcer, stricture, and tumor J Thorac Cardiovasc Surg 1963 45 13 34 14011100

[B9] Holmes RS Vaughan TL Epidemiology and pathogenesis of esophageal cancer Semin Radiat Oncol 2007 17 2 9 10.1016/j.semradonc.2006.09.003 17185192

[B10] European Cancer Observatory http://eu-cancer.iarc.fr/

[B11] Griffiths-Jones S Grocock RJ van Dongen S Bateman A Enright AJ miRBase: microRNA sequences, targets and gene nomenclature Nucleic Acids Res 2006 34 Database D140 144 10.1093/nar/gkj112 PMC1347474 16381832

[B12] Griffiths-Jones S The microRNA Registry Nucleic Acids Res 2004 32 Database D109 111 10.1093/nar/gkh023 PMC308757 14681370

[B13] Bagnyukova TV Pogribny IP Chekhun VF MicroRNAs in normal and cancer cells: a new class of gene expression regulators Exp Oncol 2006 28 263 269 17285108

[B14] Sassen S Miska EA Caldas C MicroRNA: implications for cancer Virchows Arch 2008 452 1 10 18040713 10.1007/s00428-007-0532-2 PMC2151131

[B15] Mirnezami AH Pickard K Zhang L Primrose JN Packham G MicroRNAs: key players in carcinogenesis and novel therapeutic targets Eur J Surg Oncol 2009 35 339 347 10.1016/j.ejso.2008.06.006 18644693

[B16] Calin GA Sevignani C Dumitru CD Hyslop T Noch E Yendamuri S Shimizu M Rattan S Bullrich F Negrini M Croce CM Human microRNA genes are frequently located at fragile sites and genomic regions involved in cancers Proc Natl Acad Sci USA 2004 101 2999 3004 10.1073/pnas.0307323101 14973191 PMC365734

[B17] Blenkiron C Miska EA miRNAs in cancer: approaches, aetiology, diagnostics and therapy Hum Mol Genet 2007 16 Spec No 1 R106 113 17613543 10.1093/hmg/ddm056

[B18] Kato M Slack FJ microRNAs: small molecules with big roles - C. elegans to human cancer Biol Cell 2008 100 71 81 10.1042/BC20070078 18199046

[B19] Feber A Xi L Luketich JD Pennathur A Landreneau RJ Wu M Swanson SJ Godfrey TE Litle VR MicroRNA expression profiles of esophageal cancer J Thorac Cardiovasc Surg 2008 135 255 260 10.1016/j.jtcvs.2007.08.055 18242245 PMC2265073

[B20] Maru DM Singh RR Hannah C Albarracin CT Li YX Abraham R Romans AM Yao H Luthra MG Anandasabapathy S Swisher SG Hofstetter WL Rashid A Luthra R MicroRNA-196a is a potential marker of progression during Barrett's metaplasia-dysplasia-invasive adenocarcinoma sequence in esophagus Am J Pathol 2009 174 1940 1948 10.2353/ajpath.2009.080718 19342367 PMC2671281

[B21] Mathé EA Nguyen GH Bowman ED Zhao Y Budhu A Schetter AJ Braun R Reimers M Kumamoto K Hughes D Altorki NK Casson AG Liu CG Wang XW Yanaihara N Hagiwara N Dannenberg AJ Miyashita M Croce CM Harris CC MicroRNA expression in squamous cell carcinoma and adenocarcinoma of the esophagus: associations with survival Clin Cancer Res 2009 15 6192 6200 10.1158/1078-0432.CCR-09-1467 19789312 PMC2933109

[B22] Yang H Gu J Wang KK Zhang W Xing J Chen Z Ajani JA Wu X MicroRNA expression signatures in Barrett's esophagus and esophageal adenocarcinoma Clin Cancer Res 2009 15 5744 5752 10.1158/1078-0432.CCR-09-0385 19737949 PMC2745487

[B23] Specht K Richter T Müller U Walch A Werner M Höfler H Quantitative gene expression analysis in microdissected archival formalin-fixed and paraffin-embedded tumor tissue Am J Pathol 2001 158 419 429 10.1016/S0002-9440(10)63985-5 11159180 PMC1850313

[B24] Abrahamsen HN Steiniche T Nexo E Hamilton-Dutoit SJ Sorensen BS Towards quantitative mRNA analysis in paraffin-embedded tissues using real-time reverse transcriptase-polymerase chain reaction: a methodological study on lymph nodes from melanoma patients J Mol Diagn 2003 5 34 41 10.1016/S1525-1578(10)60449-7 12552078 PMC1907376

[B25] Doleshal M Magotra AA Choudhury B Cannon BD Labourier E Szafranska AE Evaluation and validation of total RNA extraction methods for microRNA expression analyses in formalin-fixed, paraffin-embedded tissues J Mol Diagn 2008 10 203 211 10.2353/jmoldx.2008.070153 18403610 PMC2329784

[B26] Siebolts U Varnholt H Drebber U Dienes HP Wickenhauser C Odenthal M Tissues from routine pathology archives are suitable for microRNA analyses by quantitative PCR J Clin Pathol 2009 62 84 88 10.1136/jcp.2008.058339 18755714 PMC2603282

[B27] Nonn L Vaishnav A Gallagher L Gann PH mRNA and micro-RNA expression analysis in laser-capture microdissected prostate biopsies: valuable tool for risk assessment and prevention trials Exp Mol Pathol 2010 88 45 51 10.1016/j.yexmp.2009.10.005 19874819 PMC2815196

[B28] Noutsias M Rohde M Block A Klippert K Lettau O Blunert K Hummel M Kühl U Lehmkuhl H Hetzer R Rauch U Poller W Pauschinger M Schultheiss HP Volk HD Kotsch K Preamplification techniques for real-time RT-PCR analyses of endomyocardial biopsies BMC Mol Biol 2008 9 3 10.1186/1471-2199-9-3 18194512 PMC2262094

[B29] Endogenous Controls for Real-Time Quantitation of miRNA Using TaqMan^® ^MicroRNA Assays http://www3.appliedbiosystems.com/cms/groups/mcb_marketing/documents/generaldocuments/cms_044972.pdf

[B30] Vandesompele J De Preter K Pattyn F Poppe B Van Roy N De Paepe A Speleman F Accurate normalization of real-time quantitative RT-PCR data by geometric averaging of multiple internal control genes Genome Biol 2002 3 34.1 34.12 10.1186/gb-2002-3-7-research0034 PMC126239 12184808

[B31] Mestdagh P Van Vlierberghe P De Weer A Muth D Westermann F Speleman F Vandesompele J A novel and universal method for microRNA RT-qPCR data normalization Genome Biol 2009 10 R64 10.1186/gb-2009-10-6-r64 19531210 PMC2718498

[B32] R Development Core Team R: A language and environment for statistical computing 2011 R Foundation for Statistical Computing, Vienna, Austria http://www.R-project.org/ISBN 3-900051-07-0

[B33] Pfaffl MW A new mathematical model for relative quantification in real-time RT-PCR Nucleic Acids Res 2001 29 2002 2007 10.1093/nar/29.9.e45 PMC55695 11328886

[B34] O'Riordan JM Abdel-latif MM Ravi N McNamara D Byrne PJ McDonald GS Keeling PW Kelleher D Reynolds JV Proinflammatory cytokine and nuclear factor kappa-B expression along the inflammation-metaplasia-dysplasia-adenocarcinoma sequence in the esophagus Am J Gastroenterol 2005 100 1257 1264 10.1111/j.1572-0241.2005.41338.x 15929754

[B35] Colleypriest BJ Ward SG Tosh D How does inflammation cause Barrett's metaplasia? Curr Opin Pharmacol 2009 9 721 726 10.1016/j.coph.2009.09.005 19828375

[B36] Burgess JN Payne WS Andersen HA Weiland LH Carlson HC Barrett esophagus: the columnar-epithelial-lined lower esophagus Mayo Clin Proc 1971 46 728 734 5128394

[B37] Szafranska AE Doleshal M Edmunds HS Gordon S Luttges J Munding JB Barth RJ Jr Gutmann EJ Suriawinata AA Marc Pipas J Tannapfel A Korc M Hahn SA Labourier E Tsongalis GJ Analysis of microRNAs in pancreatic fine-needle aspirates can classify benign and malignant tissues Clin Chem 2008 54 1716 1724 10.1373/clinchem.2008.109603 18719196 PMC4040292

[B38] Bloomston M Frankel WL Petrocca F Volinia S Alder H Hagan JP Liu CG Bhatt D Taccioli C Croce CM MicroRNA expression patterns to differentiate pancreatic adenocarcinoma from normal pancreas and chronic pancreatitis JAMA 2007 297 1901 1908 10.1001/jama.297.17.1901 17473300

[B39] Kumarswamy R Volkmann I Thum T Regulation and function of miRNA-21 in health and disease RNA Biol 2011 8 1 8 10.4161/rna.8.1.15302 PMC3256347 21712654

[B40] Saini S Majid S Yamamura S Tabatabai ZL Suh SO Shahryari V Chen Y Deng G Tanaka Y Dahiya R Regulatory role of miR-203 in prostate cancer progression and metastasis Clin Cancer Res 2011 17 5287 5298 10.1158/1078-0432.CCR-10-2619 21159887

